# Radiographic technical quality of root canal treatment performed ex vivo by dental students at Valencia University Medical and Dental School, Spain

**DOI:** 10.4317/medoral.19176

**Published:** 2013-10-13

**Authors:** Sophie Román-Richon, Vicente Faus-Matoses, Teresa Alegre-Domingo, Vicente J. Faus-Llácer

**Affiliations:** 1 Master in Conservative Dentistry and Endodontics; 2 Associate Professor of Conservative Dentistry and Endodontics. Master Professor in Conservative Dentistry and Endodontics; 3 Professor of Conservative Dentistry and Endodontics. Master of Conservative Dentistry and Endodontics’ director. Valencia University Medical and School, Spain

## Abstract

Objectives: To evaluate radiographically the quality of root canal fillings and compare manual and rotary preparation performed on extracted teeth by undergraduate dental students. Study Design: A total of 561 premolars and molars extracted teeth were prepared using nickel-titanium rotary files or manual instrumentation and filled with gutta-percha using a cold lateral condensation technique, by 4th grade undergraduate students. Periapical radiographs were used to assess the technical quality of the root canal filling, evaluating three variables: length, density and taper. These data were recorded, scored and used to study the “technical success rate” and the “overall score”. The length of each root canal filling was classified as acceptable, short and overfilled, based on their relationship with the radiographic apex. Density and taper of filling were evaluated based on the presence of voids and the uniform tapering of the filling, respectively. Statistical analysis was used to evaluate the quality of root canal treatment, considering p < 0.05 as a statistical significant level. Results: The percentage of technical success was 44% and the overall score was 7.8 out of 10. Technical success and overall score were greater with rotary instruments (52% against 28% with a manual one, p < 0.001; 8.3 against 6.7 respectively, p < 0.001). Conclusions: It appears that inexperienced operators perform better root canal treatment (RCT) with the use of rotary instrumentation.

** Key words:**Dental education, endodontics, rotary instrumentation, radiographs, root canal treatment, undergraduate students.

## Introduction

Root canal treatment (RCT) is principally concerned with the elimination or prevention of pulpal and periapical disease (1). The purpose of root canal treatment is either to maintain asepsis of the root canal system or to disinfect it adequately (2). Root canal therapy is usually performed in three stages; first, the neurovascular tissues are removed from the root canal system. The root canal system is then shaped, in order to maintain access to apical anatomy, and finally obturated. The purpose of the root canal filling is to prevent subsequent ingress of bacteria, and re-infection of the root-canal system (1). The technical quality of a root filling has been extensively documented as an important determinant in the success of root canal treatment (3,4).

The methods used to determine the technical outcome of RCTs are based mainly on radiographical evaluation (5-7).

According to the consensus report of the European Society of Endodontology, appropriate RCT includes a radiographical control showing a prepared root canal tapered from crown to apex and filled completely without space between canal filling and canal walls. Furthermore, the root canal filling should be placed within 0.5–2 mm of the radiographical apex to prevent post-treatment disease (1).

There are clear educational guidelines regarding the experience that dental undergraduate students should ideally gain in the area of root canal treatment. The European Society of Endodontology has produced Undergraduate curriculum guidelines for endodontology; these state that new graduates should “*have a detailed knowledge of the principles and practice of non-surgical root canal treatment for vital and non-vital uncomplicated cases*” (8).

The aim of this study was to evaluate radiographically the quality of root canal fillings and compare manual and rotary preparation performed ex vivo by undergraduate students at the Valencia University Medical and Dental School, in Spain.

## Material and Methods

A total of 623 extracted teeth were scrutinized. Records that didn’t include evaluable postoperative radiographs were excluded; therefore the final sample consisted of 561 extracted teeth (295 molars and 266 premolars), representing a total of 1300 canals. All the RCTs were carried out by 4th grade undergraduate students at the Valencia University Medical and Dental School, during the 2008/09 course.

From this final sample, a total of 385 teeth (220 molars and 165 premolars) were prepared using a crown-down technique with nickel-titanium rotary files (Protaper Universal®, Dentsply Maillefer, Ballaigues, Switzerland), and the others 176 (75 molars and 101 premolars) using the step-back technique for manual instrumentation (stainless steel FlexoFile®, Dentsply Maillefer, Ballai-gues, Switzerland).

All the 561 teeth were filled using a cold lateral condensation technique (ISO standardized gutta-percha obturating points and Top Seal®, Dentsply Maillefer, Ballaigues, Switzerland).

The students were taught standardized protocol for conventional periapical radiographs (standardized measures of the gypsum cubes holding the extracted teeth, and use of beam-aligning RINN® XCP, Kodak Dental Systems, Carestream Health, Rochester, NY, USA).

The postoperative radiographs were used to assess the technical quality of the RCTs. Two investigators examined the radiographs independently using an X-ray viewer. In case of disagreement, a third investigator was asked to evaluate the radiograph and a final agreement was reached.

As in other similar studies (7,9,10), to assess the quality of canal fillings, three variables (length, density and taper) were evaluated and scored, for each canal, as follows in  Table 1.

**Table 1:**
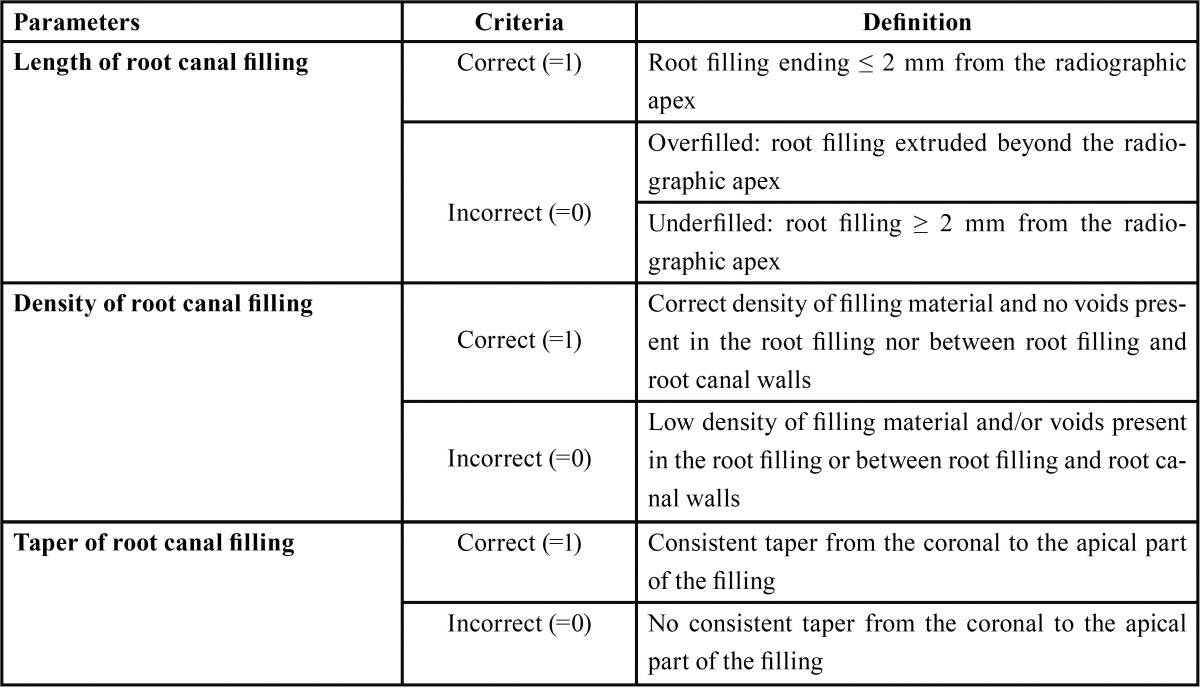
Parameters used to evaluate and score the quality of RCTs.

Besides, in multi-rooted teeth, after evaluating and scoring each root canal independently, an overall score was attributed for the tooth. To evaluate an “entire tooth” two variables were determined, based on a modification of Moussa-Badran’s criteria (9).

•TECHNICAL SUCCESS: dicotomical variable scored with ‘1’ if the three criteria were correct in all the canals of the tooth, with ‘0’ if any criteria were incorrect.

**Figure 1:**
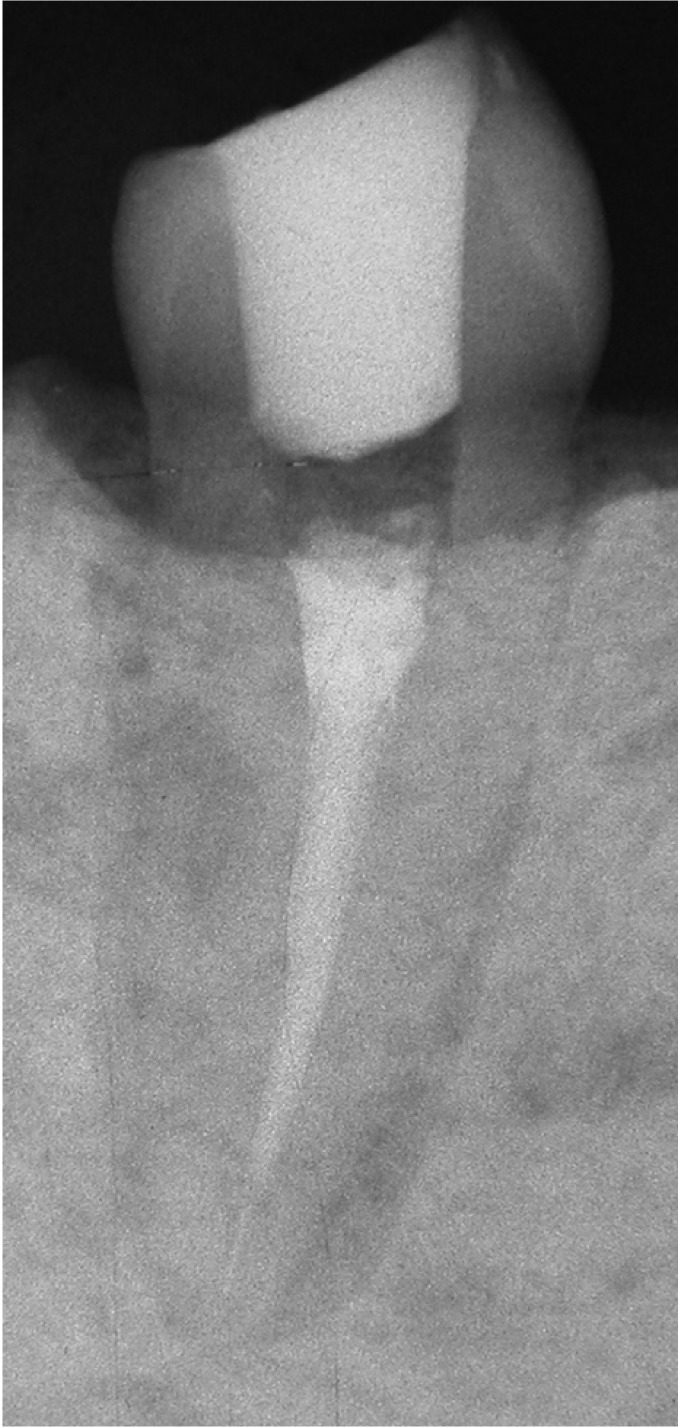
Evaluation of the tooth # 205 (premolar; 1 canal; manual instrumentation). Length = 0; Density = 0; Taper = 0 ? Overall score = 0.

**Figure 2:**
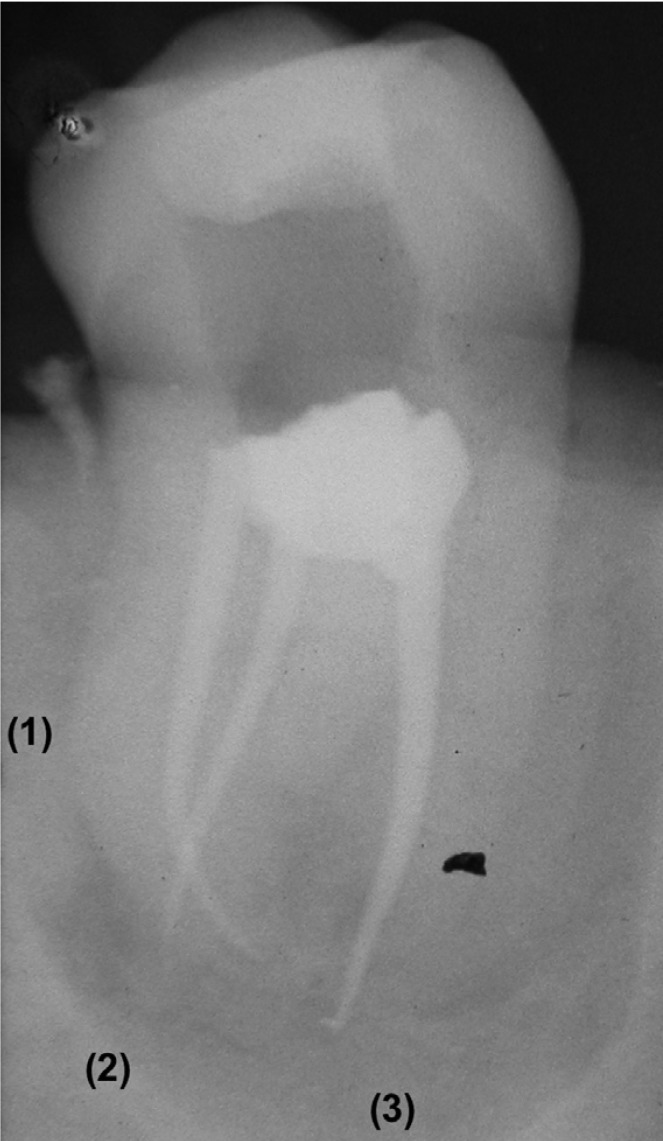
Evaluation of tooth # 248 (molar; 3 canals; rotary instrumentation). (1) (2) First and second canals: Length = 1; Density = 1; Taper = 1 (3) Third canal: Length = 0; Density = 1; Taper = 1 ? Overall score = 8.9.

•OVERALL SCORE: continuous variable scored with ‘10’ if the three criteria were correct in all the canals, and ‘0’ if all the criteria in all the canals were incorrect. This variable took intermediate scores following a lineal form (from 0 to 10), according to the number of correct criteria (Figs. 1,2).

Technical success and overall score served to study RCTs quality related to the canal fillings characteristics. It was also analysed the influence of type of tooth, number of canals and type of instrumentation.

Statistical analysis was performed with the SPSS Win Version 15.0 Package program, to determine the quality of root fillings and to investigate differences between manual and rotary preparation, using Kolmogorov-Smirnov, Kruskal-Wallis, Mann-Whitney and Chi-square analysis. The statistically significant level was p < 0.05. Observer reliability was studied using Cohen kappa test.

## Results

The overall kappa statistic for inter-examiner reliability was 0.36, the proportional agreement being 69%.

The percentage of canal fillings with correct length was 90%; correct density 69%, and with correct taper 71%. Furthermore it appears that the first criteria (length) was the best one performed by the students.

Considering the “entire teeth”, the percentage of technical success (all criteria correct) was 44% of the sample; and the overall score average was 7.8 over 10.

According to tooth type, it was shown that the technical success obtained for premolars was superior than the one reached for molars (52% for premolars vs. 38% for molars; p < 0.001). Considering the variable “overall score”, the difference was not significant (7.8 and 7.6 respectively; p = 0.06) but it was shown that there was at least a tendency.

The comparison of technical success rates according to the number of canals showed lower results as the number of canals increased (1 canal: 66%; 2 canals: 41%; 3 canals: 38% and 4 canals: 33%; p < 0.001). Similar results were obtained with the variable “overall score” (1 canal: 8.2; 2 canals: 7.6; 3 canals: 7.7 and 4 canals: 7.2; p = 0.004).

Focusing on type of instrumentation, the percentage of technical success was highly improved with rotary instrumentation (52% vs. 28% with manual one; p < 0.001) and the overall score average, considering the whole sample, was superior than the one obtained with manual instrumentation (8.2 and 6.7 respectively; p < 0.001).

This was true for both molars and premolars, but this effect was even more apparent when the treated tooth was a molar. For molars, the difference of technical success between both instrumentations was 29% (45% of technical success with rotary instrumentation vs. 16% with manual one) and for premolars this difference was 23% (60% and 37% respectively), with p < 0.001. For overall score averages the results were similar (molars: 8.2 and 6.1; premolars: 8.3 and 7.1 respectively; p < 0.001).

Furthermore it could be observed that the use of rotary instrumentation was more advantageous as the number of canals increased. In teeth with 2 or 3 canals the rotary instrumentation permitted to reach higher technical success (2 canals: 51% with rotary instrumentation and 24% with manual one; 3 canals: 46% and 14% respectively; p < 0.001). The same trend was shown for “overall score” variable (2 canals: 8.2 with rotary instrumentation and 6.6 with manual one; 3 canals: 8.2 and 6.1 respectively; p < 0.001).

In teeth with 1 or 4 canals, the rotary instrumentation was still superior to the manual one but the difference was not so apparent (1 canal: technical success of 69% for rotary instrumentation vs. 59% for manual one, and overall score of 8.4 vs. 8.0 respectively. 4 canals: technical success of 44% for rotary instrumentation vs. 16% for manual one and overall score of 8.0 vs. 6.0 respectively).

## Discussion

The inter-examiner reliability (0.36) may be taken to represent poor agreement, even if beyond chance (11). This data shows that, as advised by Saunders et al. in 2000 (12), further effort should have been taken to calibrate the observers.

Studies published in the international literature have shown that the technical standards of root canal treatments completed by general dental practitioners are often less than ideal (13-15). There are various possible reasons why this is so. Criticism has often been directed at educational sources, highlighting a lack of adequate educational exposure at an undergraduate level (16).

In this study 44% of the 561-teeth sample were considered to be technically correct and the overall score average was 7.8 over 10.

Although it is difficult to compare the data because of the different criteria used, other similar works looked into the quality of RCTs performed by undergraduate dental students: in France, Moussa-Badran et al. found that 30.1% of the studied RCTs had adequate quality (9); in Jordan, 47.4% (10) and in Turkey, 33% of the root fillings had correct technical quality (7). These three studies report results similar to the ones presented in this work; the following others two showed different success rates: in Wales, only 13% of root fillings were categorized as satisfactory (17), and in Ireland, 70% of root fillings placed in single rooted teeth were within 2 mm of the radiographic apex (18).

The European Society of Endodontology recommends that undergraduate endodontic teaching should be supervised by specialists or by staff with a special knowledge and interest in endodontics (8). The professors in charge of the endodontic teaching during the fourth grade at the Valencia Dental School are qualified specialists and the staff:students ratio is above 1:12.

According to the European Society of Endodontology, another important criteria is the time allocated to the subject; in concrete terms, in this faculty, 150 hours are devoted to preclinical endodontic teaching (8).

Comparing these data to the information obtained by Qualtrough et al. at an international level in 1999 (19), it appears that the time allocated to preclinical endodontic teaching at the Valencia Medical and Dental School during the fourth grade is between the highest but the staff:students ratio could be improved.

Many studies have yet demonstrated the efficiency and advantages of rotary instrumentation; it provides an easier way to shape the canal root maintaining the original curvature and avoiding zips (20-25); it also permits to realize faster treatments (20,21,23-25). However, its limitations should also be underlined; comparing with manual preparation, rotary instrumentation presents a higher risk of file separation (21,22).

With this study it was shown that inexperienced operators performed better RCTs using rotary instrumentation; moreover another work published in 2003 by Sonntag et al. presented similar conclusions, although they used manual Ni-Ti files instead of stainless steel ones (26). But as explained by Georgelin-Gurgel et al. in a study published in 2008, it is very important that undergraduate students can acquire skill in use of rotary instrumentation, receiving specific preclinical training in order to avoid iatrogenic events, such as file separation or loss of working length (27).

## Conclusions

The percentage of technically successful RCTs was 44% and the overall score average of the sample was 7.8 over 10.

A relevant point of this study is that, despite the poor general results, both technical success rate and overall score were highly improved when the preparation was carried out with rotary instrumentation. It can be concluded that rotary instrumentation was in general more adequate than manual one and especially efficient in molars. Thus, it appeared that inexperienced operators performed better RCTs with the use of rotary instrumentation.
